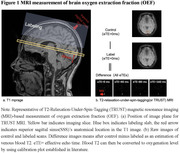# Hypertension‐Induced Accelerating Brain Oxygen Extraction Fraction Precedes Age‐Related White Matter Dysfunction in Asymptomatic Cognitive Decline

**DOI:** 10.1002/alz.095265

**Published:** 2025-01-09

**Authors:** Yifan Yan, Xuhao Zhao, Yaping Zhang, Wanxin Li, Zhiying Lin, Yi Zhou, Shenghao Fang, Jingkai Huang, Peige Song, Zixuan Lin, Xin Xu

**Affiliations:** ^1^ School of Public Health, the Second Affiliated Hospital of School of Medicine, Zhejiang University, Hangzhou, Zhejiang China; ^2^ The Key Laboratory of Intelligent Preventive Medicine of Zhejiang Province, Hangzhou, Zhejiang China; ^3^ Key Laboratory for Biomedical Engineering of Ministry of Education, Department of Biomedical Engineering, College of Biomedical Engineering &Instrument Science, Zhejiang University, Hangzhou, Zhejiang China; ^4^ Memory, Ageing, and Cognition Centre (MACC), Department of Pharmacology, Yong Loo Lin School of Medicine, National University of Singapore, Singapore, Singapore Singapore

## Abstract

**Background:**

Oxygen extraction fraction (OEF) reflects the equilibrium between brain oxygen delivery and consumption, potentially serving as an early Alzheimer’s disease (AD) biomarker. Previous investigations were mainly conducted in cognitive impairment and AD population. However, the potential neuropathway connecting cardiometabolic condition, OEF, and dementia‐related brain structural changes, especially in early cognitive decline, is unclear. This study investigated the associations of OEF with white matter dysfunction (WMD) among cognitively normal older adults and explored OEF’s mediating role between early cardiometabolic conditions and WMD.

**Method:**

Eligible cognitively intact older participants (aged above 50 years without cognitive impairment on a comprehensive cognitive battery) were recruited from communities in China and completed the demographic, medical investigations and MRI scans. OEF was measured using T2‐relaxation‐under‐spin‐tagging (TRUST). WMD was assessed by two certified neuroradiologists from T2‐flair images to obtain a Fazekas score. Subjective cognitive decline (SCD) was assessed. Cardiometabolic conditions, including hypertension, diabetes mellitus, hyperlipidemia, stroke, and heart disease were assessed through medical history and/or lab measurements. General linear regression models and formal mediation analysis were performed. Sensitivity analysis was performed among participants without any cardiovascular diseases (stroke and/or heart disease).

**Result:**

Among a total of 267 individuals (mean age 68.4±8.8 years, 183(68.5%)female, 118(44.2%) SCD), OEF ranged from 28.6% to 53.5% (IQR = 5.3). After adjustment for demographics (age, sex, education), lifestyle factors (smoke, BMI, and physical inactivity), medical history, and SCD status, increasing OEF was associated with worsen Fazekas scores (β = 0.02, 95%CI = (0.01,0.04)). Both hypertension (β = 1.90, 95%CI = (0.53,3.27)) and stroke (β = ‐2.54, 95%CI = (‐4.42,‐0.67)) were independently associated with OEF. Mediation analysis demonstrated that higher OEF mediated the effects of hypertension on Fazekas scores (mediation effect = 0.04, 95%CI = (0.01,0.09)). The mediation proportion was up to 13.0% (p = 0.02). Robust mediation effect was found among participants without any cardiovascular diseases.

**Conclusion:**

Abnormal brain oxygen delivery is associated with WMD at the early stage of cognitive impairment. Our study elucidates the potentially casual influence of OEF on the pathway between hypertension and WMD. OEF could be utilizable as a fast and convenient measurement in predicting early brain structural disturbances implicated in cognitive impairment and dementia.